# Artemisinin-Based Combination Therapy Synergized with Medicinal Plants to Induce Musculotoxic Effects

**DOI:** 10.1155/2021/8861574

**Published:** 2021-07-08

**Authors:** Thérèse Daubrey-Potey, Valéry Adjogoua, Mamadou Kamagaté, Serges Aoussi, Mireille Dosso

**Affiliations:** ^1^Clinical Pharmacology Department, Faculty of Medical Sciences, Félix Houphouet-Boigny University, BP V 34 Abidjan 01, Abidjan, Côte d'Ivoire; ^2^Institut Pasteur of Côte-d'Ivoire, BP 450 Abidjan 01, Abidjan, Côte d'Ivoire; ^3^Clinical Pharmacology Department, Faculty of Medical Sciences, Alassane Ouattara University Bouaké, BP V 1801, Bouaké, Côte d'Ivoire

## Abstract

**Introduction:**

Multivisceral, neurological, hepatic, and renal damage has been witnessed following the use of artemisinin-based combination therapy (ACT) and herbal medicine. These multiple organ damages make us think of muscle damage. The objective was to study the myotoxicity of the combination of ACTs with medicinal plants.

**Materials and Methods:**

Muscle cells (RD cells) were brought into contact with preparations of antimalarial drugs and/or antimalarial herbs. The following drugs were used: artesunate 100 mg/amodiaquine 270 mg (ASAQ) and artemether 80 mg/lumefantrine 480 mg (AL); plant *Sida acuta* (PSA) and plant *Enantia polycarpa* (PEP) at 10 *µ*g/ml. After 5 days of incubation, the cells were counted by using a hemocytometer with trypan blue solution.

**Results:**

Artesunate/amodiaquine caused a significant drop in the number of muscle cells, compared to the control, between D2 and D4 (*p* < 0.001). There was also a significant difference between the control and artemether/lumefantrine between D2 (*p* < 0.01) and D4 (*p* < 0.001) and between the control and the *Sida acuta* plant, on D2 (*p* < 0.001), D4 (*p* < 0.001), and D5 (*p* < 0.05). In tubes treated with ASAQ and *Sida acuta*, cell mortality was over 30%. Finally, statistically significant cell destruction in the tubes treated with the combination of antimalarial drugs and traditional plants compared to those of the control was observed from D2 (*p* < 0.001).

**Conclusion:**

Artemisinin-based combination therapy remains effective and well tolerated. But its combination with medicinal plants induced myotoxic effects. This toxicity would appear to be of the additive type. Further studies should be able to better elucidate the mechanism of this toxicity.

## 1. Introduction

The World Health Organization (WHO) recommends the use of therapeutic combinations, especially those based on artemisinin derivatives (ACTs). Nowadays, they are widely used worldwide, mainly in areas of chloroquine resistance and resistance to artemisinin monotherapy [1]. The advent of these combination therapies seemed to be a lifeline. The aim was to improve the efficacy of antimalarials through a synergistic effect of associated molecules in order to delay the onset of drug resistance. Although this effectiveness remains undeniable in Africa and particularly in Sub-Saharan Africa, we noted an upsurge in serious adverse effects linked to their use. In 2005, its frequency was estimated at 57% in Cote d'Ivoire [2].

Thus, these adverse drug reactions which were reported to the pharmacovigilance unit of the Pharmacology Department of the Faculty of Medical Sciences in Abidjan (Cote d'Ivoire) and to the Ivorian National Pharmacovigilance Center were serious and unexpected. They were cases of blackwater fevers, hepatitis, and hepatonephritis with high mortality involving antimalarial drugs, in particular, ACTs [3]. Monomorphic and polymorphic injuries represented 49% and 51% of cases, respectively [4]. Isolated or multiple neurological, hepatic, and renal damages were noted [5, 6]. In front of these multiple organ damages, we suspect muscle damage explaining the myalgia and stiffness or general muscle aches encountered, which seem unrecognized. The mechanisms of occurrence are unclear. The constant risk factors for the occurrence of these adverse reactions are self-medication, drug abuse or misuse, and concomitant combinations of antimalarials and/or traditional therapy [3]. However, in West Africa, the use of traditional medicine and medicinal plants for primary health care is common [7]. The objective of this study was to assess the toxicity of the combination of ACTs with antimalarial plant extracts on muscle cells.

## 2. Materials and Methods

Aqueous plant extracts and drug preparations (ACTs) have been made to be in contact with muscle cells. Muscle cells served as an experimental model.

### 2.1. Cell Lines

The musculocytes or myocytes used were RD muscle cells from rhabdomyosarcoma. These muscle cells were obtained from CDC, Atlanta. They were continuous heteroploid lines, which, due to their rapid growth, were readily available and widely used. The cells were thawed and cultured in T75 culture dishes (75 cm^2^) on enriched MEM medium. The MEM culture medium is enriched with 10% decomplemented foetal calf serum (FCS), 1% mixture of antibiotics (10 IU/ml of penicillin, 10 mg/ml of streptomycin; Sigma Aldrich), and 1% of L-glutamine (Sigma Aldrich) for cell growth.

### 2.2. Products Used

The antimalarial drugs used were artesunate 100 mg/amodiaquine 270 mg (ASAQ) and artemether 80 mg/lumefantrine 480 mg (AL). The antimalarial plant extracts used were *Sida acuta* (PSA), *Malvaceae* and *Enantia polycar*pa (PEP), *Annonaceae* obtained from the medicinal plant market in Abidjan. Their efficacy had previously been demonstrated [8–10]. Samples of the bark of *Enantia polycarpa* and the leafy stem of *Sida acuta* were used in our study. Trypan blue from Sigma Aldrich was also used to identify living or dead cells.

### 2.3. Cell Culture

The cells were trypsinized. 0.5.10^5^ cells/ml was cultured in 2 ml of 10% MEM growth medium in 51 T25 culture tubes (25 cm^2^) and incubated for 48 h at 36.5 ± 0.5°C under 5% CO₂. Then, the growth medium was emptied.

### 2.4. Preparation of Plants

*Enantia polycarpa* bark samples were thoroughly washed and dried. These samples were crushed into small pieces and then ground with the use of a suitable grinder to obtain a very fine powder. *Enantia polycarpa* bark powder (200 g) obtained was added to two liters (2 L) of distilled water. A magnetic stirrer allowed the mixture to be stirred for 24 hours. Then, the resulting solution was filtered three times through cotton wool and Whatman paper. The solution was oven-dried at 40°C. The powder thus obtained constituted the aqueous extract of powdered bark of *Enantia polycarpa*.

A preparation of the aqueous extract of the leafy stems of *Sida acuta* was made. Two hundred grams (200 g) of leafy stems of *Sida acuta* were washed and put in 2 L of distilled water. The mixture was boiled for 15 minutes. The resulting solution was cooled and filtered three times through cotton wool and Whatman paper and then oven-dried at 40°C. The powder obtained was the aqueous extract of the leafy stems of *Sida acuta*.

### 2.5. Preparation of Drugs

The artesunate 100 mg/amodiaquine 270 mg (ASAQ) and artemether 80 mg/lumefantrine 480 mg (AL) tablets were crushed. The resulting powder was suspended in 2% MEM.

### 2.6. Study of Cellular Toxicity

A concentration of 1 mg/ml of all the products tested has been prepared. This concentration was reduced to 10 *μ*g/ml by means of two-tenth dilutions. Thus, in the T25 culture dishes, 5 ml of the plant and drug preparations were brought into contact with the RD muscle cells. Artemether/lumefantrine, artesunate/amodiaquine, *Sida acuta*, and *Enantia polycarpa* were first tested one by one. Then, the products were tested 2 by 2 by combining antimalarials with extracts of medicinal plants. The toxicity of each product was tested in a batch of 5 dishes (5 boxes per product) compared to a batch of 5 negative control dishes (no product was added). Each combined test (ASAQ + PSA, ASAQ + PEP, AL + PSA, AL + PEP) was carried out in a batch of 5 dishes and a batch of 5 dishes serving as control or negative control. After inoculation, the dishes were placed in an oven at 36.5 ± 0.5°C under 5% CO₂. Cells were observed on daily basis for 5 days under an inverted fluorescence microscope for cellular carpet confluence. The effect of antimalarials and traditional herbs on muscle cells was measured on daily counting of the number of living and dead cells by using a hemocytometer. Before counting cell, trypan blue was added (dilution factor 2). 30 *µ*L of the mixture (trypan blue + cell suspension) was sampled to fill up the 2 chambers of the hemocytometer by gently touching the edge of the coverslip with the cone. We enumerated separately viable nucleated cells and dead nucleated cells. For authentication, viable or living nucleated cells appear clear, nonblue, and dead nucleated cells appear blue because of damaged cell membrane.

According to Coulerie [11], a product is considered to be cytotoxic when it causes cell mortality greater than 30% at a concentration of 10 *µ*g/ml. The experiments were carried out in triplicate. In this preliminary study, we could have coupled the first method with Cell Counting Kit-8 (CCK-8). It is a sensitive colorimetric assay for the determination of cell viability in cell proliferation and cytotoxicity assays. It could have allowed us to validate our experience. But, the lack of financial support did not allow us.

### 2.7. Statistical Analysis

Data were estimated as mean with standard deviation (M* *± SD) of *n* experiments. Statistical analysis of data was performed by using GraphPad Instat software (Microsoft, San Diego, California, USA) and GraphPad Prism software (Microsoft, San Diego, California, USA). Statistical analyses were assessed by using one-way or two-way analysis of variance (ANOVA) followed by Bonferroni's posttest when applicable. Statistical significance was considered at *p* < 0.05.

## 3. Results

### 3.1. Effects of Monotherapies on Myocytes

**Figure 1:**
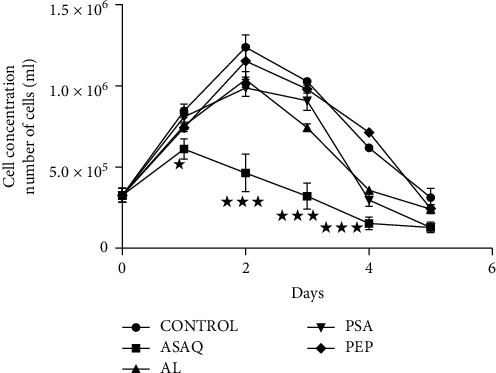
Effect of monotherapies on myocyte concentration.

Figure 1 showed the effect of antimalarials and herbal extracts on muscle cells compared to a cell control. Artesunate/amodiaquine caused a significant drop in the number of myocytes, compared to that of the control, between D2 and D4 (*p* < 0.001). A significant difference was also noted between the control and artemether/lumefantrine between D2 (*p* < 0.01) and D4 (*p* < 0.001), and between the control and the *Sida acuta* plant, on D2 (*p* < 0.001), D4 (*p* < 0.001), and D5 (*p* < 0.05).

Data are given as means ± SEM of 3 different experiments. Statistical analyses were assessed using two-way analysis of variance (ANOVA) followed by Bonferroni's posttest ^*∗*^*p* < 0.05, ^*∗∗*^*p* < 0.01, and ^*∗∗∗*^*p* < 0.001 versus control.

Table 1 gives the percentage of deaths of the myocytes in the various tubes treated with the products. The myocyte mortality associated with the use of artesunate/amodiaquine was over 30% from D2 to D5. According to Coulerie, this indicated that the product was toxic. Regarding *Sida acuta* plant, mortality was also over 30% at D4 and D5. This indicated its cellular toxicity at D4 and D5. Low cell mortality was observed with the plant *Enantia polycarpa*.

### 3.2. Effects of Combination Therapies on Myocytes

**Figure 2:**
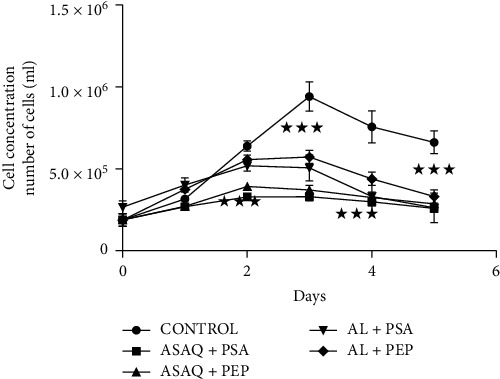
Effect of combination therapies on myocyte concentration.

We compared the effect of the combination of antimalarial drugs and herbal extracts versus a control on myocytes (Figure 2). All the curves looked alike. But we noticed a statistically significant cell destruction in the tubes treated with the combination of antimalarial drugs and extracts of medicinal plants compared to the control tube from D2 (*p* < 0.001) for associations with artesunate/amodiaquine and from D3 (*p* < 0.001) for combinations with artemether/lumefantrine. The destruction was more massive in the combinations containing artesunate/amodiaquine.

Data are given as means ± SEM of 3 different experiments. Statistical analyses were assessed using two-way analysis of variance (ANOVA) followed by Bonferroni's post-test ^*∗∗∗*^*p* < 0,001 versus control.

Table 2 indicated the percentage of cell death in the tubes treated with the products. The myocyte mortality in the combinations containing artesunate/amodiaquine was over 30% from D2. That of combinations containing artemether/lumefantrine was over 30% from D3. The combination of antimalarials and traditional plants could therefore be considered toxic. Higher mortality was observed for combinations containing artesunate/amodiaquine. In this case the toxicity was more pronounced.

## 4. Discussion

**Figure 3:**
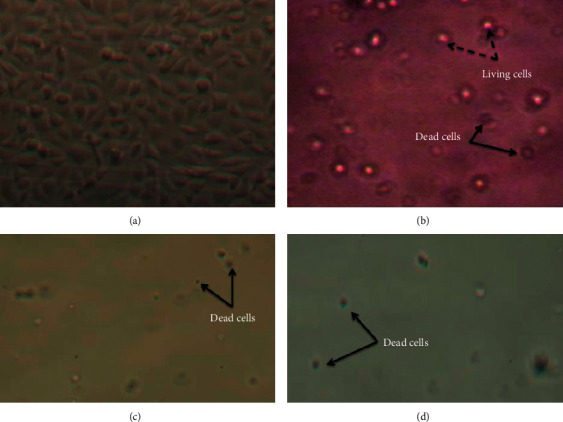
Muscle cells (RD cells) in culture under microscope (magnification 10 × 120). (a) Living muscle cells in culture at D0; (b) living ( ) and dead muscle cells ( ) treated with 10 ug/ml of *Sida acuta* (PSA) at D2; (c) dead muscle cells treated with 10 ug/ml of *Sida acuta* (PSA) at D5; (d) dead muscle cells treated with 10 ug/ml of artesunate/amodiaquine at D5.

Artesunate/amodiaquine, artemether/lumefantrine and the plant *Sida acuta* showed muscle cell toxicity or musculotoxicity. However, in monotherapy artesunate/amodiaquine and the plant *Sida acuta* appeared to be the most musculotoxic (Figure 3). Musculotoxicity of 4-aminoquinolines has been reported with chloroquine and hydroxychloroquine [12–17]. After treatment for a few weeks to a few years, myalgia gradually sets in with muscle weakness followed by muscle atrophy. It was accompanied by a change in sensory sensitivity, loss of the tendon reflex, as well as an abnormality in nerve conduction related to neuropathy. This was reversible when treatment was dropped or discontinued [12, 13]. Lysosomal dysfunction with vacuolar accumulation of metabolic products was believed to play a role in muscle toxicity of 4-aminoquinolines [17]. Indeed, muscle damage could be related to the amphiphilic properties of these drugs especially chloroquine and hydroxychloroquine like amodiaquine, leading to specific lysosomal disruptions and autophagic dysfunctions [15]. This possibility of muscle damage could also explain the worsening of malaria symptoms such as asthenia, myalgia or muscle stiffness or soreness, and muscle weakness merging with neurological disorders according to the summary of product characteristics. Neuromyopathy and extrapyramidal syndromes were described with artesunate/amodiaquine [18].

Muscular disorder with artemether-lumefantrine has also been described. Kamagaté et al. [19] reported two cases of polyneuropathy included myopathies, due to artemether-lumefantrine. These polyneuropathies were characterized by muscular disorders such as flask paralysis without superficial sensitivity disorder. Furthermore, with regard to lumefantrine toxicity, it has been reported that, in combination with artemether, adverse reactions commonly included arthralgia, myalgia, fatigue, muscle contraction, and muscle cramps. This could probably explain muscle stress and muscle lysis [18].

In combination therapy with plant, artesunate/amodiaquine and artemether/lumefantrine had comparable average musculotoxicity. The plants appeared to enhance the muscle toxicity of conventional antimalarials. Additive effect was observed. This musculotoxicity could be close to the hematologic, nervous, and renal toxicity of the combination of antimalarial drugs and traditional plants. Indeed, in previous studies [5, 6], it has been shown that the combination of antimalarial drugs and traditional plants would lead to hematologic toxicity, renal toxicity, and nerve toxicity. These findings would allow to understand that the strong muscular lyses or rhabdomyolysis could lead in certain cases to renal failure observed during the treatment of malaria with high ASAT/ALAT ratio. In several studies on the serious adverse effects of antimalarials, a combination of antimalarial drugs and traditional plants has been found [3, 4, 19]. Thus, the study of Die-Kacou et al. [20] showed that a combination of ACT with traditional plant was responsible for the occurrence of hepatitis, hepatonephritis, and blackwater fevers with renal failure. Patients should then avoid the combination of conventional antimalarials with traditional therapy. Indeed, this combination would increase the risk of hematologic, renal, nerve, and muscle toxicity. The findings of this study suggest that the biological monitoring of malaria treatment could include the determination of muscular creatine phosphokinase (CPK) in renal failure or myalgia or deep asthenia.

## 5. Conclusions

Although in medical practice, ACT for malaria treatment remains effective and well tolerated, the present study showed myotoxic effects of artesunate/amodiaquine, *Sida acuta*, and combination of medicinal plants with artemisinin-based combination therapy. This toxicity would appear to be additive type. Combination of traditional plants with conventional treatment should then be avoided by patients or carefully monitored. Further studies will elucidate the mechanism of this toxicity by determining blood CPK level and anatomopathological examination.

## Figures and Tables

**Table 1:** Effect of monotherapies on myocyte mortality.

Products	Days of incubation (D)	Mean ± SD					
		0	1	2	3	4	5
MEM 2%	0.3	0.1	0.1	0.5	0.2	0.3	0.24 ± 0.17
ASAQ	0.3	34	67.97	76.98	81.96	51.16	62.41 ± 19.74^*∗*^
AL	0.3	11.74	16.70	28.70	28.60	12.76	19.70 ± 8.38
PSA	0.3	8.78	20.58	17.40	58.35	53.19	31.66 ± 22.50
PEP	0.3	18.06	5.99	5.39	0	13.88	8.66 ± 7.22

ASAQ: artesunate/amodiaquine; AL:artemether/lumefantine; PSA:plant of *Sida acuta*; PEP: plant of *Enantia polycarpa*; SD = standard deviation; Statistical analyses were assessed using one-way analysis of variance (ANOVA) followed by Bonferroni's posttest ^*∗*^*p* < 0,05 versus control.

**Table 2:** Effect of combination therapies on the mortality of myocyte (%).

Products	Days of incubation (D)	Mean ± SD					
		0	1	2	3	4	5
MEM 2%	1.6	0.17	0.26	0.1	0.09	0.23	0.17 ± 0.08
ASAQ + PSA	1.6	12.89	40.33	59.76	54.20	58.68	45.17 ± 19.64^*∗*^
ASAQ + PEP	1.6	7.75	36.49	55.41	53.14	69.08	44.37 ± 23.52^*∗*^
AL + PSA	1.6	13.55	13.17	30.89	44.73	47.81	30.03 ± 16.50
AL + PEP	1.6	7.48	11.05	32.13	32.83	50.85	26.87 ± 17.78

ASAQ: artesunate/amodiaquine; AL: artemether/lumefantine; PSA: plant of *Sida acuta*; PEP: plant of *Enantia polycarpa*; SD = standard deviation. Statistical analyses were assessed using one-way analysis of variance (ANOVA) followed by Bonferroni's posttest ^*∗*^*p* < 0,05 versus control.

## Data Availability

The data used to support the findings of this study are available from the author upon request.
